# Strain-Dependent Photoacoustic Characteristics of Free-Standing Carbon-Nanocomposite Transmitters

**DOI:** 10.3390/s22093432

**Published:** 2022-04-30

**Authors:** Muhammad Faraz, Muhammad Awais Abbasi, Donghee Son, Changhwan Shin, Kyu-Tae Lee, Sang Min Won, Hyoung Won Baac

**Affiliations:** 1 Department of Electrical and Computer Engineering, Sungkyunkwan University, Suwon 16419, Korea; mfaraz@skku.edu (M.F.); awais@skku.edu (M.A.A.); daniel3600@skku.edu (D.S.); 2 School of Electrical Engineering, Korea University, Seoul 02841, Korea; cshin@korea.ac.kr; 3 Department of Physics, Inha University, Incheon 22212, Korea

**Keywords:** photoacoustic transmitter, nanocomposites, strain sensor, stretchable device

## Abstract

In this paper we demonstrate strain-dependent photoacoustic (PA) characteristics of free-standing nanocomposite transmitters that are made of carbon nanotubes (CNT) and candle soot nanoparticles (CSNP) with an elastomeric polymer matrix. We analyzed and compared PA output performances of these transmitters which are prepared first on glass substrates and then in a delaminated free-standing form for strain-dependent characterization. This confirms that the nanocomposite transmitters with lower concentration of nanoparticles exhibit more flexible and stretchable property in terms of Young’s modulus in a range of 4.08–10.57 kPa. Then, a dynamic endurance test was performed revealing that both types of transmitters are reliable with pressure amplitude variation as low as 8–15% over 100–800 stretching cycles for a strain level of 5–28% with dynamic endurance in range of 0.28–2.8%. Then, after 2000 cycles, the transmitters showed pressure amplitude variation of 6–29% (dynamic endurance range of 0.21–1.03%) at a fixed strain level of 28%. This suggests that the free-standing nanocomposite transmitters can be used as a strain sensor under a variety of environments providing robustness under repeated stretching cycles.

## 1. Introduction

A significant interest has been devoted to the development of stretchable devices for their utilization of strain-dependent characteristics in biomedical applications, stretchable chemical sensors, flexible circuits, electrical interconnects, transistor matrix and displays [1,2,3,4,5,6,7,8,9,10,11,12]. Such property would be highly beneficial for ultrasonic devices and their applications such as structural health monitoring, medical diagnosis, and non-destructive evaluation (NDE). However, conventional ultrasonic technologies typically employ rigid piezoelectric transducers that are fabricated on hard substrates. With lack of flexibility and robustness, these are not suitable for strain-dependent applications. Moreover, it is difficult to make a conformal interface between transducer and object whose surface is rough, irregular, or non-homogeneous [13]. Only a few examples have realized stretchable piezoelectric ultrasound transducers for applications to NDE of internal defects, in which an object has a non-planar complex surface [14]. However, these systems experienced an issue of partial delamination of electrical contacts when stretched by 30–40%. Moreover, as their monolithic piezoelectric layer loses its thickness uniformity, these can be exposed to noisy environment in a time domain, also resulting in unstable frequency characteristics due to the breakage of thickness resonance.

Photoacoustic (PA) transmitters without electrical connections have attained a considerable attention owing to their characteristic advantages in terms of strong output pressure amplitude, high operation frequency easily going up to hundreds of MHz, and broad bandwidths. Moreover, their output waveform can have a clear single pulse with a minimal ringing tail, which is generated with a long pulse-to-pulse interval (e.g., tens of ms). These transmitters have been fabricated in a nanocomposite form by mixing carbon nanoparticles into polydimethylsiloxane (PDMS) as an elastic polymer matrix with the high coefficient of thermal expansion (0.92 × 10^−3^ K^−1^). Carbon-based nanoparticles, including carbon nanotube (CNT), CNT yarn, carbon black, carbon nanofibers, and candle soot nanoparticles (CSNP), have been widely used as high-efficiency PA sources owing to their physical dimension, low specific heat capacity, rapid heat transition, and high optical absorption [15,16,17,18,19,20,21,22,23,24,25,26,27,28,29,30]. PDMS has been extensively employed as an elastomer in various chemical and biological applications as well as in stretchable interconnects, flexible conductive patterns, and lab-on-a-chip systems due to its distinctive properties such as optical transparency, chemical stability, biocompatibility, and mechanical flexibility [31,32,33,34,35,36,37,38,39].

Carbon nanocomposites have also been employed to develop a variety of sensors. For example, CNT-PDMS composite-based piezoresistive sensors were operated via electric resistance variation of the nanocomposite structure when subject to strain or deflection [38,40,41]. However, for carbon nanocomposites working as PA transmitters, strain-dependent PA characteristics in terms of pressure amplitude and frequency responses have not been investigated in detail, which are useful to determine strain sensitivity, linear measurement range, and detection limit [29,42]. These significantly depend on the mixture weight ratios of carbon nanoparticles in composite. This weight ratio also determines the rigidity and flexibility of the nanocomposites [38,40,41]. In addition, reliability and endurance of carbon nanocomposite films needs attention for comprehensive analysis, in terms of PA signal variation depending on strain and stretching cycles [43].

As the performance of CSNP and CNT based PA transmitters under similar conditions have not been reported in detail, therefore we compared the performance of CSNP and CNT based PA transmitters prepared by utilizing similar fabrication process and under similar experimental conditions. Moreover, strain-dependent PA characteristics of both the nanocomposites have not been investigated in detail.

We demonstrate strain-dependent characterization of CNT- and CSNP-PDMS PA transmitters. First, we present the fabrication process of PA transmitters and their characterization in glass-bound form. Next, we characterize the strain-dependent PA outputs for free standing nanocomposite transmitters. The transmitters are compared in terms of output pressure waveform, frequency response, strain-dependent PA performance, and dynamic endurance (E). Then, the flexible and stretchable properties of free-standing PA transmitters are characterized in terms of Young’s modulus. Mechanical durability and reliability of PA transmitters is also evaluated by performing a dynamic endurance test (*DET*) under various strain values and repeated stretching conditions. Finally, laser-induced damage threshold is investigated to quantify the mechanical robustness of the PA transmitters.

## 2. Materials and Methods

### 2.1. Transmitter Fabrication

**Figure 1:**
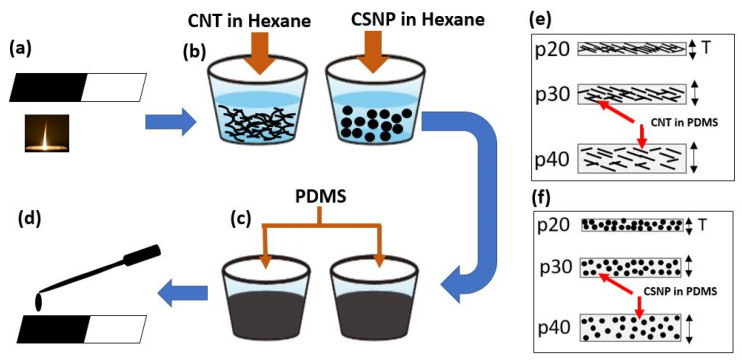
(**a**–**d**) Stepwise fabrication details to develop carbon nanoparticle-based PA transmitters. (**a**) Collection of CSNP from candle flame, (**b**) Mixing CNT and CSNP in hexane and sonicating for 2 h, (**c**) Adding PDMS to sonicated solution, (**d**) Drop-casting the prepared solution on glass substrate, (**e**,**f**) A simplified schematic to represent nanoparticle concentration and thickness (T) of CNT and CSNP transmitters respectively. The transmitters p20, p30, and p40 correspond to the PDMS-to-carbon nanoparticle ratios of 20:1, 30:1, and 40:1, respectively.

Figure 1 depicts the fabrication process of the PA transmitters described elsewhere [29]. The solutions of CNT (multi-walled CNT with a cylindrical shape of 1–2 µm in length and 10–20 nm in diameter [44]) and CSNP in PDMS were prepared by utilizing a homogeneous dispersion method. The CSNP were harvested on a glass slide from a paraffin wax candle (35-mm diameter) by using a flame synthesis process [45]. These uniformly deposited CSNP were carefully transferred into a glass bottle. Then, CNT and the CSNP were separately mixed with hexane in a 1:40 (carbon nanoparticles: hexane) weight ratio to obtain CNT-hexane and CSNP-hexane solutions. Both solutions were sonicated for 2 h to improve dispersion of carbon nanoparticles in hexane. Then, a mixture of PDMS prepolymer liquid and its curing agent (Sylgard 184, Dow Corning, Midland, MI, USA) prepared in a 10:1 weight ratio was further mixed with the CNT-hexane and CSNP-hexane solutions. Each ratio of CNT and CSNP to PDMS was adjusted to 1:20 weight ratio, respectively (named CNT p20 and CSNP p20). The hexane lowers the viscosity of the PDMS and ensures uniform blending of CNT and CSNP in PDMS. Two planar transmitters were fabricated by drop-casting each solution (one for CNT-hexane-PDMS and the other for CSNP-hexane-PDMS) onto a glass substrate. Then, the transmitters were cured at 90 °C for 30 min. Similarly, we also prepared two more solutions for each of CNT and CSNP with different PDMS ratios (1:30 and 1:40). For all these solutions, the weight ratio of PDMS to hexane was 1:2 (PDMS: hexane). Each transmitter was denoted as p20, p30, or p40 according to the ratio of PDMS to CNT and CSNP, respectively. The optical extinction was >99% for all the transmitters.

**Figure 2:**
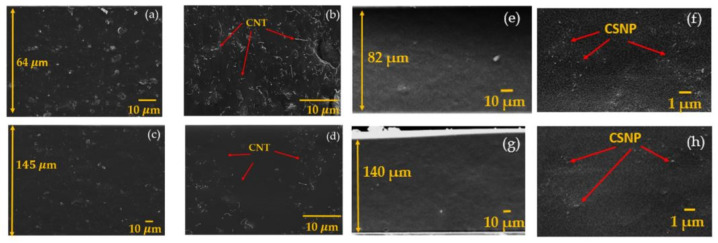
Scanning electron microscopic images of CNT-PDMS and CSNP-PDMS composite films (p20 and p40). (**a**–**d**) Cross-sectional and its enlarged views for CNT p20 in (**a**,**b**) and CNT p40 in (**c**,**d**). (**e**–**h**) Similar images for CSNP p20 in (**e**,**f**) and CSNP p40 in (**g**,**h**). The images of CSNP-PDMS (p40) composite film were reproduced from [29].

The cross-sectional images of the fabricated CNT-PDMS composites were obtained by utilizing a scanning electron microscope (SEM) (JSM 7000F, JEOL, Tokyo, Japan) (Figure 2). The entire cross-sections of the composite CNT p20 and p40 presented in Figure 2a,c confirm uniform distribution of CNT in space. The enlarged views are also depicted in Figure 2b,d, respectively. The entire composite film thickness of CNT p20 (64 µm) was relatively thinner than that of CNT p40, while its concentration of nanoparticles was higher. For CSNP-PDMS composites used for comparison, their SEM images were similar with those previously reported in [29]. The film thickness for CSNP p20 and CSNP p40 were 82 and 140 µm, respectively.

### 2.2. PA Characterization of Nanocomposite Transmitters on Glass Substrate

The PA transmitter output was characterized by using an experimental setup similar with [29]. A *Q*-switched 7-ns pulsed laser (Nd:YAG Litron Laser, Rugby, UK) was used as an excitation source with 532-nm wavelength and 10-Hz pulse repetition rate. The input laser beam (8-mm diameter) was first mitigated by employing neutral density filters, and then incident onto the transparent glass side (a PA composite layer on the other side). A polyvinylidene difluoride (PVDF) needle-type hydrophone (Precision Acoustics, Dorchester, UK; 1-mm diameter, 6-dB bandwidth of 20 MHz) was utilized to detect PA outputs generated by the transmitters. The detector was placed at 2-mm distance apart from the PA transmitter, satisfying a plane-wave incidence configuration. The pressure amplitude detected by the PVDF hydrophone was then monitored by using a digital oscilloscope (WaveSurfer 452, LeCroy, New York, NY, USA). The temporal PA waveforms were acquired by averaging 20 signal traces. For characterization of mechanical robustness, the laser-induced damage threshold was determined by using the same setup reported in [29], following the measurement procedure [18].

### 2.3. Strain-Dependent PA Characterization of Free-Standing Nanocomposite Transmitters

**Figure 3:**
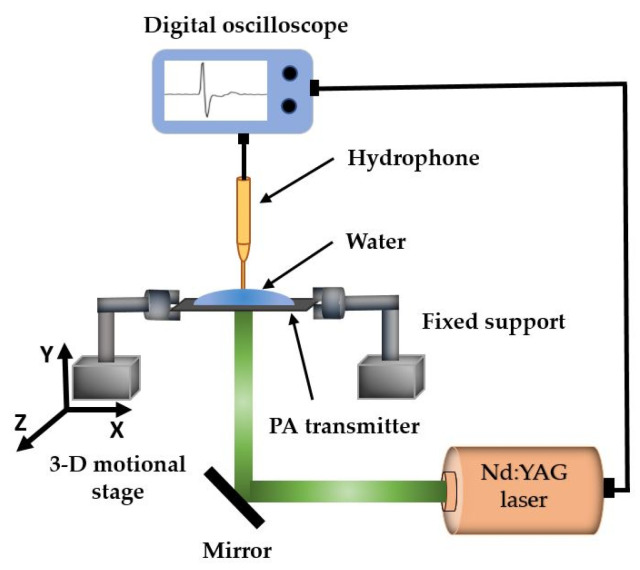
Experimental setup to determine PA output pressure amplitudes under an external strain applied to the free-standing transmitter not bound to a glass substrate.

Strain-dependent PA outputs for CNT- and CSNP-PDMS transmitters were characterized by using the setup shown in Figure 3. The laser beam was irradiated from the bottom, whereas the acoustic signal was detected by the needle hydrophone from the top side of transmitter. For measurement, the transmitter was initially fixed between two grips, each of which was connected with a separate support. One of the supporting arms was fixed, while the other was connected to the motional stage. The longitudinal strain was applied by moving the arm in x-direction only. The PA pressure was then measured by using the detector which is coupled with water on the top surface of the transmitter as shown in Figure 3.

## 3. Results and Discussion

### 3.1. PA Characteristics of Nanocomposite Transmitters on Glass Substrates

**Figure 4:**
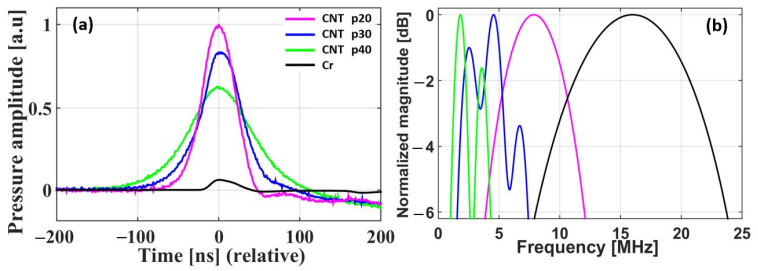
(**a**) Temporal output waveforms and (**b**) their frequency spectra for CNT p20, p30, p40, and Cr film.

First, the CNT-PDMS composite transmitters were characterized with comparison to a reference Cr film and also CSNP-PDMS transmitters previously reported in [29]. The output pressure waveforms generated by the CNT-PDMS composites (p20–p40) were measured by using the laser energy of 1.5 mJ/pulse. The temporal waveforms are shown in Figure 4a. The CNT-based p20 composite transmitter generated the highest output pressure signal among them: 16.67 times (24.4 dB) higher than that of Cr film, and also 1.6 and 4.4 dB higher than those produced by CNT p30 and p40, respectively. The decrease in the pressure amplitude with the increased PDMS ratio agrees with previous results. The output amplitude of CNT p20 was also slightly higher than those of the CSNP-based transmitters (p20–p40) reported in [29] by 1.4, 2.4, and 4.4 dB respectively.

Figure 4b shows the frequency spectra of the temporal waveforms presented in Figure 4a, including the bandwidth effect of detector. From CNT p20 to p40, the center frequency was decreased from 7.87 to 1.82 MHz due to the thickness of optical absorption and the increased PDMS ratio. Furthermore, the 6-dB bandwidth was reduced from 8.13 to 3.30 MHz; for CNSP-PDMS transmitters previously reported, the center frequency was decreased from 3.90 (p20) to 2.44 MHz (p40), and 6-dB bandwidth from 7.17 (p20) to 5.80 MHz (p40), respectively [29].

In thin-film transmitters, a temporal pressure output is obtained by taking the convolution of a temporal profile of incident laser pulse and a temporal profile converted from the spatial depth of optical absorption (time = distance/sound speed). This means that the increase in thickness of nanocomposite film results in pulse broadening, shift in center frequency to a lower range (also due to acoustic attenuation through the increased thickness), and a relatively narrow frequency bandwidth. Thus, with the increase in film thickness, the center frequency and 6-dB bandwidth shifted from 7.87 to 1.82 MHz and 8.13 to 3.30 MHz.

We prepared transmitters with different ratios of PDMS to carbon nanoparticles. With the increase in ratio of PDMS to carbon nanoparticles, we obtained film thickness from 64 to 145 µm. As expected by the above theoretical background, the thinnest composite p20 (64 µm) (highest concentration of nanoparticles and smallest optical absorption depth) produces the highest peak pressure output as compared to p30 and p40 (Figure 4a). On the other hand, the thickest composite p40 (145 µm) (lowest concentration of nanoparticles and highest optical absorption depth) exhibits the lowest peak pressure value. Thus, the p20 composite generates the highest output pressure signal as compared to the other two transmitters discussed here.

### 3.2. Strain-Dependent PA Characteristics of Free-Standing Nanocomposite Transmitters

The strain dependence of the transmitters was characterized with respect to concentration of the carbon nanoparticles and thickness of the free-standing composite films. Using the experimental setup shown in Figure 3, we measured the strain-dependent PA pressure (*P*) and frequency response of each transmitter. Then, the performance was evaluated in terms of stress (*σ*) and strain (*S*) applied to each free-standing composite film after delamination from the glass substrate. Moreover, for comparison, free-standing CSNP-based nanocomposite films were similarly prepared and used for performance evaluation.

**Figure 5:**
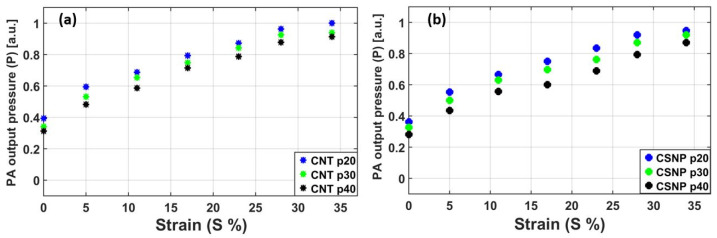
PA output pressure amplitudes measured under various strain conditions for the free-standing nanocomposite transmitters with different nanoparticle concentrations: (**a**) CNT-PDMS, (**b**) CSNP-PDMS.

Figure 5 shows the enhancement of peak pressure amplitudes obtained from both types of free-standing nanocomposite films under the application of strain; CNT-PDMS for Figure 5a and CSNP-PDMS for Figure 5b. Each dot in Figure 5 represents the peak amplitude of each PA waveform. All the amplitudes in both figures were normalized to the highest value obtained with CNT p20. The transmitters were stretched by a 1-mm step up to 24 mm (initially 18 mm without strain). The maximum-applied longitudinal strain was 0.34 to avoid physical damage or breakage of the free-standing transmitters.

For all nanocomposite films, the PA pressure amplitude increased with the strain from *S* = 0 (without strain) to 34%. Moreover, the higher concentration of carbon nanoparticles generated the stronger output pressure for a fixed strain. In comparison of both types of nanocomposite transmitters, the PA amplitude of the CNT-based nanocomposite was slightly stronger than that of the CSNP case with the same concentration. For *S* = 0 (no strain) and 34% (maximum strain), the output pressure amplitudes by CNT p20 were 0.72 and 0.47 dB higher than those of CSNP p20, respectively. This provides a linearized average slope (*L_P_*) defined by a PA output increment per strain variation (*L_P_* = Δ*P*/Δ*S*). For CNT p20 and CSNP p20, *L_P_* was 1.84 and 1.77 together with percentage variation in each pressure amplitude of 154 and 161%, respectively from *S* = 0 to 34%. The output pressure amplitude by CNT-based transmitters was found to be slightly higher than those of the corresponding CSNP transmitters both in the glass bound and in free-standing composite form. This can be attributed to non-uniform dispersion characteristics and agglomeration effects of CSNP particles within the PDMS matrix which may alter thermal relaxation time, resulting in slightly lower performance of CSNP composite transmitters.

**Figure 6:**
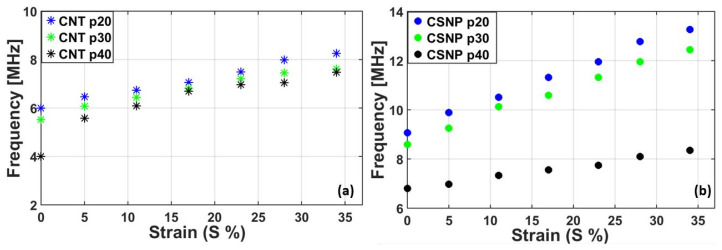
Center frequencies for the PA pressure outputs shown in Figure 5. (**a**) CNT-PDMS, (**b**) CSNP-PDMS.

Using the PA pressure outputs shown in Figure 5, we obtained their center frequencies as shown in Figure 6. At the maximum strain of *S* = 34% applied to CNT p20, the center frequency was increased from 6 to 8.26 MHz. This is due to the tightly packed nanoparticles in the composite, resulting in the reduced absorber thickness under the strain. The other transmitters also showed similar increasing trends: CNT p30 from 5.52 to 7.61 MHz, and CNT p40 from 4 to 7.48 MHz. For the CNSP cases, the center frequency enhancement was more pronounced: CSNP p20 from 9.0 to 13.27 MHz, CSNP p30 from 8.6 to 12.5 MHz, and CSNP p40 from 6.8 to 8.35 MHz. A linearized average slope defined for the center frequency *L_f_* = Δ*f*/Δ*S* for CNT p20 and CSNP p20 was 6.8 and 12.6 MHz/mm over a range from *S* = 0 to 34%, respectively. Our analysis shown in Figure 5 and Figure 6 suggests that the PA transmitters can respond sensitively to the externally applied strain in terms of peak pressure amplitudes and center frequencies.

**Figure 7:**
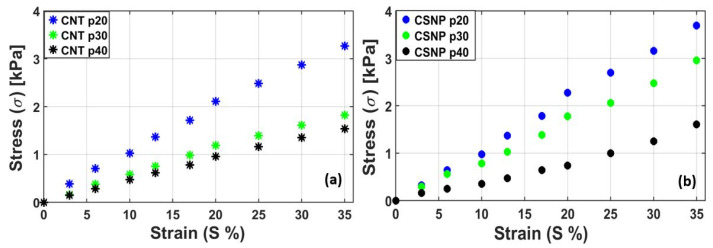
Stress–strain curves for the free-standing nanocomposites: (**a**) CNT-PDMS and (**b**) CSNP-PDMS.

Then, we performed an experiment to evaluate a stress–strain relationship for all the free-standing nanocomposite transmitters to determine Young’s modulus. Figure 7a,b show the stress–strain relationships for CNT-PDMS and CSNP-PDMS composites, respectively. For both cases, the slopes (i.e., Young’s modulus = Δ*σ*/Δ*S*) increased with the nanoparticle concentration [40,46]. For the CNT-based nanocomposites, the total stress endured by CNT p20, p30, and p40 at *S* = 0.35 was 3.26, 1.82, and 1.54 kPa, respectively. Moreover, the CSNP p20, p30, and p40 had 3.69, 2.95, and 1.61 kPa, respectively, for the same measurement condition. Note that the increased nanoparticle concentration leads to stronger particle-particle (carbon nanoparticles are intertwined together) and particle-PDMS interaction, thus forming more rigid transmitters. We also observed that the CSNP-based nanocomposites (p20–p40) demonstrated slightly higher or similar values in Young’s modulus (10.57, 8.50, and 4.56 kPa) as compared to those of the corresponding CNT-based composites (10.48, 5.61, and 4.08 kPa).

### 3.3. Dynamic Endurance Test (DET)

For the dynamic endurance evaluation, each free-standing nanocomposite transmitter was subject to a cyclic test with repeated expansion and contraction (ESM 1500, Mark 10, New York, NY, USA). All the nanocomposites were subject to strain values of 5, 17, and 28% by placing each composite film between two grips of the test stand. For each strain value, the transmitters were tested with 100, 200, 400, 600, and 800 number of cycles (*N*). Once each test cycle was completed (e.g., 5% strain with 100 cycles), then the change in PA output pressure was measured again with the applied strain (e.g., 5%) by utilizing the setup shown in Figure 3. The obtained PA output pressure (*P_a_*) was normalized with respect to *P*_0_ (PA pressure at *S* = 0).

**Figure 8:**
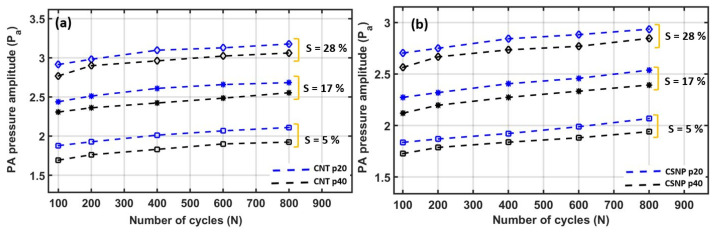
*DET* results for carbon-nanocomposite transmitters. (**a**) CNT-based nanocomposite transmitters, (**b**) CSNP-based nanocomposite transmitters (*S*: strain).

Figure 8a,b show the *DET* results for CNT-PDMS and CSNP-PDMS composites, respectively. Here, when *S* = 5%, the PA pressure output for CNT p20 was initially increased by 24.7% for *N* = 100 as compared to the PA pressure obtained at a similar strain of 5% in Figure 5a. The PA output after 100 cyclic motions was then increased by 12% when *N* = 800 (the lowest blue curve with square markers in Figure 8a). Similarly, when *S* = 5% in case of CSNP-based transmitters, the output PA pressure of CSNP p20 (the lowest blue curve with square marker) was increased by 20.4% for *N* = 100 cycles as compared to the initial pressure in Figure 5a and then again increased by 12% from *N* = 100 to 800 as shown in Figure 8b. For the other cases (*S* = 17 and 28%) in Figure 8a,b, the PA pressure amplitude variations were in the range of 8–13% from *N* = 100 to 800. After stretching cycles from *N* = 100 to 800, the pressure variation under a given strain can be described as a dynamic endurance (*E*) defined here as a percentage change in *P_a_* divided by *S*. Low *E* means better endurance of the PA output against an applied strain.

The *DET* results are summarized for 5, 17, and 28% strain in Table 1. The DET results demonstrate that the free-standing nanocomposite transmitters provide reliable PA outputs, with *E* values in range of 0.28–2.8% after such cyclic expansion and contraction.

**Figure 9:**
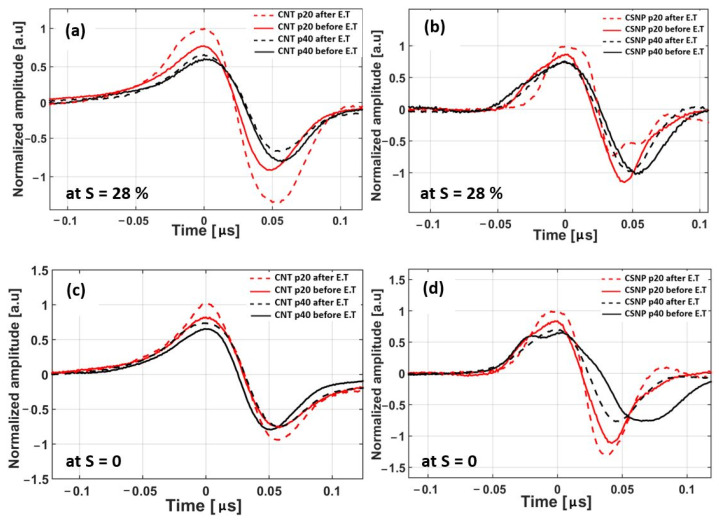
Comparison of time-domain PA waveforms before and after DET. (**a**) CNT-PDMS transmitters at *S* = 28%, (**b**) CSNP-PDMS transmitters at *S* = 28%, (**c**) CNT-PDMS transmitters at *S* = 0, and (**d**) CSNP-PDMS transmitters at *S* = 0. Note that the waveforms were measured first setting *S* = 28% in (**a**,**b**), and then measured again setting the strain back to *S* = 0 in (**c**,**d**).

We compared the time-domain PA waveforms before and after DET (*S* = 28% and *N* = 2000). The waveforms in Figure 9a were normalized to that of CNT p20 after DET, and the waveforms in Figure 9b to that of CSNP p20 after DET. After the DET in this harsh condition, the peak amplitudes of CNT p20 and p40 were increased by 29 and 10%, with the corresponding *E* of 1.03 and 0.35%, respectively. Similarly, for CSNP p20 and p40, the pressure amplitudes increased by 16 and 6% with *E* = 0.57 and 0.21%, respectively.

In order to validate the reliability of the free-standing nanocomposite transmitters, the *DET* was then performed again by setting the strain condition back to *S* = 0% (un-stretched) with *N* = 2000 (Figure 9c,d show the pressure amplitudes normalized with respect to those of CNT p20 and CSNP p20, respectively, measured at *S* = 0 after 2000 cycles). The pressure variations for CNT-based nanocomposites (p20 and p40) were 22 and 13% with *E* = 0.78 and 0.46%. Note that the transmitters experienced the maximum strain of *S* = 28%, before measuring the pressure amplitude at *S* = 0. Hence, in this case the change in pressure amplitude was divided by *S* = 28%, instead of *S* = 0. Similarly, for the CSNP cases (p20 and p40), the pressure variations were 19 and 8% with *E* = 0.67 and 0.28%, respectively.

Figure 9 reveals that the higher the rigidity of the nanocomposite film, the higher the percentage increase in the PA pressure amplitudes and *E* of the transmitters. Thus, p20 (CNT and CSNP) transmitters showed an increased variation in pressure amplitude as well as *E* as compared to those of the corresponding p40 transmitters. Such variations can be attributed to the change in the spacing and rearrangement of the carbon nanoparticles within the PDMS caused by the applied strain. With the application of strain, carbon nanoparticles experienced a tensile stress along the direction of the applied strain and a compressive stress perpendicular to the axis of the strain. Hence, each cyclic stretching and relaxation causes the nanoparticles to buckle in plane into waves, resulting in the increase pressure amplitude. Lipomi et al. previously reported a similar phenomenon of variation in resistance (71%) of CNT-PDMS composite with the application of strain (50%) [47]. It was also reported that a change in resistance value of 113% was recorded in a CNT-PDMS-conductive pattern for a tensile strain of 41.9% [31]. Similarly, the stretchable electrodes demonstrated resistance variation of ~15% under a strain level of 20 and 25% for *N* = 2000 cycles [43]. The CNT-PDMS composite presented in [41] showed 35% variation in resistance when subject to a strain value of 20% for only 5 cyclic stretching tests. The CNT-PDMS composite (10% CNT in a weight ratio) provided a resistance change of ~75% for a strain value of 10% with 11 stretching cycles [40]. Moreover, stretchable charge-trap memory transistors exhibited malfunctions after 2000 stretching cycles under a strain level of 50% [48]. However, our proposed transmitters presented pressure amplitude variation in the range of 6–29% for a strain level of 28%. We also established that the transmitter with lower concentration of carbon nanoparticles (p40) within the PDMS matrix are more flexible and capable of producing output pressure amplitudes with variation as low as 6–13% together with *E* variation in the range of 0.21–0.46% after 2000 cycles. Therefore, the transmitters with lower concentration of carbon nanoparticles exhibited more stability and reliability against cyclic stretching with slight variation in output pressure amplitude and *E*. Thus, they can be potentially used for strain-dependent applications.

### 3.4. Mechanical Robustness against Input Optical Fluence

**Figure 10:**
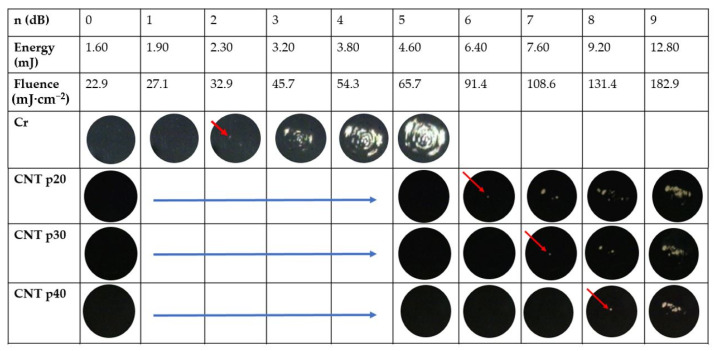
Microscopic images of CNT-based transmitters and Cr film (reference) after laser pulse excitation. Input laser energy and fluence are shown in the second and third row, respectively. The red arrows indicate the initiation point of ablation for each transmitter. The ablation was not observed for CNT p20–p40 when the optical fluence was lower than 65.7 mJ·cm^−2^ (images not shown).

Although the output pressure from PA transmitters is directly proportional to the input laser fluence and optical absorption by the nanocomposites [49], the laser-induced damaged threshold (*F_th_*) provides information about the maximum extent to which the input laser energy can be increased. Hence, the maximum-attainable PA pressure for a fixed optical absorption is determined with *F_th_*. Figure 10 shows the microscopic images of the transmitters after laser pulse excitation. While the reference Cr film has the low level of damage threshold at a pulse energy *E_th_* = 2.30 mJ and thus *F_th_* = 32.9 mJ·cm^−2^, the transmitter CNT p20 had *E_th_* = 6.40 mJ and *F_th_* = 91.4 mJ·cm^−2^. *F_th_* for CNT p20, p30, and p40 was almost 2.8, 3.3, and 4.0 times higher than that of the reference Cr film, respectively. For the CNT p30 and p40 nanocomposite transmitters, *F_th_* was 108.6 and 131.4 mJ·cm^−2^, respectively. In comparison to CSNP-based transmitters reported in [29], the higher laser-induced damage threshold by 21–39% was observed in the CNT-based nanocomposite cases.

## 4. Summary

The strain-dependent PA performances of solution-processed CNT-PDMS and CSNP-PDMS transmitters were characterized in terms of output pressure amplitude and frequency response. For characterization, the transmitters were fabricated on glass substrates and in a free-standing form. Transmitters with lower concentration of nanoparticles were found to be more flexible and stretchable with Young’s modulus in the range of 4.08–10.57 kPa. For robustness test, the dynamic endurance of transmitters was measured, exhibiting their mechanical durability and reliability by low variation of PA output pressure of 8–15% with the endurance *E* of 0.28–2.8% over 100–800 cycles for the strain value *S* of 5–28%. After 2000 cycles, the pressure amplitude variation increased to 6–29% (*E* = 0.21–1.03%) at *S* = 28%. Following this measurement with *S* = 28%, we set the un-stretched state (*S* = 0) again and obtained pressure variation of 8–22% (*E* = 0.28–0.78%), which is still similar to the case when the transmitters were stretched maximally (*S* = 28%). We expect that the strain-dependent PA responses and the dynamic endurance of the free-standing nanocomposite transmitters can be used to develop a strain sensor which has non-contact and non-electrical assessment, flexibility, and stretchability.

## Figures and Tables

**Table 1:** Dynamic *E* summary for the free-standing nanocomposite transmitters under applications of various strain value.

Applied Strain (%)	Dynamic Endurance (*E*) (%) (Pressure Variation between *N* = 100 and 800 per Strain)			
	CNT p20	CNT p40	CSNP p20	CSNP p40
5	2.4	2.8	2.4	2.4
17	0.58	0.64	0.64	0.76
28	0.28	0.35	0.28	0.35

## Data Availability

The study did not report any data.
